# Extensive unusual lesions on a large number of immersed human victims found to be from cookiecutter sharks (*Isistius* spp.): an examination of the Yemenia plane crash

**DOI:** 10.1007/s00414-016-1449-6

**Published:** 2016-09-13

**Authors:** Agathe Ribéreau-Gayon, Carolyn Rando, Yves Schuliar, Stéphane Chapenoire, Enrico R. Crema, Julien Claes, Bernard Seret, Vincent Maleret, Ruth M. Morgan

**Affiliations:** 1 Department of Security and Crime Science – Centre for the Forensic Sciences, University College London, 35 Tavistock Square, London, WC1H 9EZ UK; 2 Institute of Archaeology, University College London, 31-34 Gordon Square, London, WC1H 0PY UK; 3 Forensic and Criminal Intelligence Agency of the French Gendarmerie, 5, boulevard de l’Hautil, 95300 Pontoise, France; 4 Centre Hospitalier Universitaire, Pôle Odontologie et Santé Buccale, Service de Médecine Bucco-Dentaire, GHP, place Amélie Rabat-Léon, 33076 Bordeaux, France; 5 Department of Archaeology & Anthropology, Division of Archaeology, University of Cambridge, Downing Street, Cambridge, CB2 3DZ UK; 6 Marine Biology Laboratory, Earth and Life Institute, Université catholique de Louvain, Kellner building, 3, Place Croix du Sud - bte L7.06.04, 1348 Louvain-la-Neuve, Belgium; 7 Ichtyo Consult, 6 bis rue du Centre, 91430 Igny, France; 8 Maison de Santé de Blaye, 1 rue Nicole Girard-Mangin, 33390 Blaye, France

**Keywords:** Aircraft accident, Postmortem examination, Scavenging, Cookiecutter sharks, Forensic decomposition, Drowned bodies

## Abstract

Accurate determination of the origin and timing of trauma is key in medicolegal investigations when the cause and manner of death are unknown. However, distinction between criminal and accidental perimortem trauma and postmortem modifications can be challenging when facing unidentified trauma. Postmortem examination of the immersed victims of the Yemenia airplane crash (Comoros, 2009) demonstrated the challenges in diagnosing extensive unusual circular lesions found on the corpses. The objective of this study was to identify the origin and timing of occurrence (peri- or postmortem) of the lesions.A retrospective multidisciplinary study using autopsy reports (*n* = 113) and postmortem digital photos (*n* = 3 579) was conducted. Of the 113 victims recovered from the crash, 62 (54.9 %) presented unusual lesions (*n* = 560) with a median number of 7 (IQR 3 ∼ 13) and a maximum of 27 per corpse. The majority of lesions were elliptic (58 %) and had an area smaller than 10 cm^2^ (82.1 %). Some lesions (6.8 %) also showed clear tooth notches on their edges. These findings identified most of the lesions as consistent with postmortem bite marks from cookiecutter sharks (*Isistius* spp.). It suggests that cookiecutter sharks were important agents in the degradation of the corpses and thus introduced potential cognitive bias in the research of the cause and manner of death. A novel set of evidence-based identification criteria for cookiecutter bite marks on human bodies is developed to facilitate more accurate medicolegal diagnosis of cookiecutter bites.

## Introduction

**Fig. 1:**
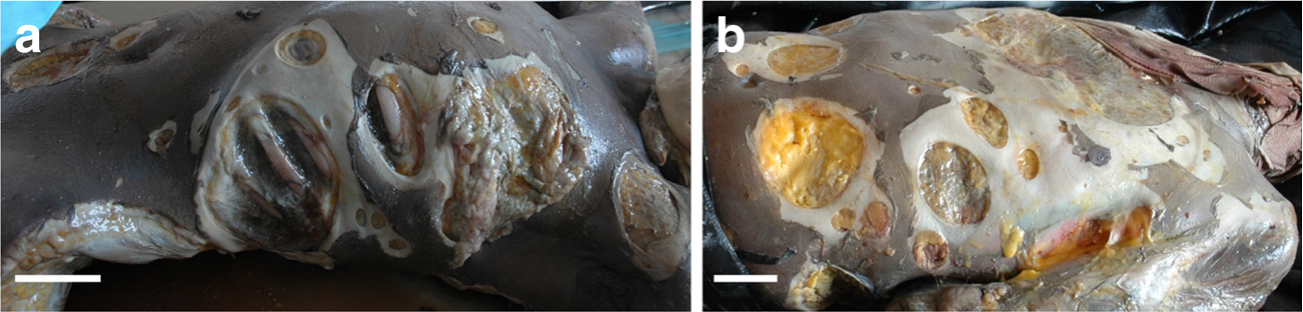
Extensive lesions of various shapes and sizes on victims of the Yemenia plane crash. Image credit: IRCGN, 2009. The figure shows the anterior surface of **a** a female and **b** a male victim of the Yemenia plane crash, extensively covered with scooped out lesions. The lesions show a great diversity in macroscopic appearance, shape, depth, and size. *Scale bars* in **a** and **b**, 5 cm

Medicolegal investigation of mass fatality disasters is highly challenging because of the large number of victims involved, dangerous environments, limited resources, social pressure and time constraints [1–16]. The investigation is all the more complex when aquatic environments are involved (i.e. natural disasters, boating accidents and aircraft crashes in the sea [6, 9, 11, 13, 15]), not only due to the technical difficulties of accessing the scene and locating victims and remains but also because aquatic taphonomic factors may deeply contribute to the degradation of corpses and thereby introduce uncertainty in establishing the cause and manner of death [2–18]. A major issue is the current lack of useable standards to reliably assess decomposition and identify postmortem modifications—including those caused by fauna—in aquatic milieu and specifically in marine environments [19–22]. Medicolegal experts can therefore face trauma of undetermined cause that hamper postmortem conclusions and their subsequent interpretation (postmortem submersion interval (PMSI), cause and manner of death, victim identity). This study focuses on the Yemenia plane crash in the Indian Ocean (30 June 2009), a case that shows how complex postmortem investigation of victims recovered from marine environments can be. In that particular case, postmortem examination of the victims revealed extensive unusual circular wounds on most of the corpses (Fig. 1a, b), the origins of which were not identified in the first medicolegal reports. This study therefore aimed to determine the origins and timing (peri- or postmortem) of the lesions, an imperative step in determining the cause and manner of death.

## Materials and methods

### Materials

On 30 June 2009, an Airbus A310 of Yemenia Airlines (flight IY26) en route from Sanaa (Yemen) to Moroni (Comoros) crashed in the Indian Ocean while approaching Moroni at 1:51 a.m. local time [23]. The accident caused 152 deaths, with only one survivor [23]. One hundred thirteen victims were recovered from two sites: (i) Comoros (*n* = 89; PMSI = 60 days) and (ii) Tanzania (*n* = 24; PMSI = 7 days). It was determined that most victims perished from polytrauma (69 %), while others died either from undetermined causes (26 %) or from polytrauma but with the possibility of drowning unable to be excluded (5 %). These proportions of causes of death are consistent with published cases of deaths from aircraft crashes [1, 24, 25]. Data concerning the crash and the victims were analysed from administrative investigation reports, autopsy file reports (*n* = 113), autopsy photos (*n* = 3 579) and underwater videos of the plane wreck (hours = 322) provided by the relevant authority (The Forensic and Criminal Intelligence Agency of the French Gendarmerie). All data were anonymised in the analyses performed in this study.

### Methods

#### Macroscopic analysis

A descriptive retrospective approach was conducted to investigate the characteristics of the unidentified lesions found on the victims. Amongst the 3579 autopsy photos available, only those showing clearly visible and measurable lesions (i.e. the flattest lesions regarding camera position with a scale located in the same plane than the lesion) were selected (*n* = 165; 4.6 %) to enable macroscopic evaluation of the lesions [26–28]. Five qualitative parameters were first recorded: (i) type of lesion (clearly discernible or superimposed/confluent), (ii) presence of tooth notches on edges of lesion (iii) substrate of lesion i.e. the nature of tissues at the bottom of lesion (dermis and hypodermis; adipose tissue and/or superficial layer of muscle; tendons, ligament, muscle and/or cortical bone), (iv) shape of the lesion outline, and (v) macroscopic appearance of lesion (fresh or aged appearance of the substrate, neat or blunt edges). The morphology of the lesions was further characterised using four quantitative parameters (area, perimeter, major axis, and ratio of major axis/minor axis, used as a proxy for shape) that were digitally measured and calculated from the autopsy photos by using ImageJ, an open-source software developed to analyse photographs in medical contexts [29]. The landmark-based method of lesion measurement was validated by an intra-class correlation coefficient test (ICC 1.0) [30]. The two observers (a hospital emergency physician and a graduate student in forensic anthropology) had comparable experience with ImageJ software and they independently measured the same set of 20 randomly selected autopsy photos.

#### Statistical analysis

Statistical analysis of the dataset was carried out using the programme R, Version 3.2.1 [31]. First, normality of the distributions of the number of lesions per corpse, area, perimeter, major axis, and ratio of major axis/minor axis was tested with a Shapiro-Wilk test. The hypothesis of a normal distribution was rejected for each variable that was either multimodal or positively skewed (number of lesions per corpse 0.9; area 4.7; perimeter 1.8; major axis 1.8; ratio of major axis/minor axis −0.3). Given the skewness of the distributions, the median is reported as summary statistics. Frequency of lesions by sexes—only for the victims for which sex was determined at postmortem analysis—was tested with a Kolmogorov-Smirnov test. Chi-square (*χ* ^2^) tests were performed to analyse the frequencies of type of lesion and tooth notches on edges of lesion. Silverman tests [32] were run to look for any multimodality in the distributions of area and ratio of major axis/minor axis of lesions, indicative of potential groups of characteristics (i.e. a ratio of major axis/minor axis of one corresponds to a geometrical circle), hence of potential different origins of the lesions. The distribution of area was log-transformed to enable performing statistical tests that require a normal distribution as an assumption. Correlation between area and substrates of lesions was researched performing a Kendall’s Tau-b test as well as a one-way ANOVA (area log-transformed) with a *post hoc* Tukey honest significant difference (HSD) test. Correlation between area and ratio of major axis/minor axis was analysed with a Kendall’s Tau-b test. A *p* value of less than 0.05 was considered as significant.

## Results

### Victims with lesions

Of the 113 victims recovered, 62 possessed unidentified circular lesions (54.9 %) (Table 1). Victims with lesions included 37 males (59.7 %), 21 females (33.9 %) and 4 of undetermined sex (6.5 %), with a male to female sex ratio of 1.8. Males and females showed no difference in the median number of lesions per corpse—respectively, 8 (IQR 4 ∼ 15.5) and 8 (IQR 3.5 ∼ 11)—(Kolmogorov-Smirnov test: *D* = 0.1609, *p* = 0.879). Information on the victims for each recovery site is presented in Table 1. A total of 560 lesions were recorded, with frequency ranging from one to 27 per corpse (IQR 3 ∼ 13) and a median number of 7 lesions per corpse (Table 2).

**Table 1:** Frequency of lesions

	Entire sample		CMR		TNZ	
	*n*	%	*n*	%	*n*	%
Victims recovered	113	100	89	78.8	24	21.2
Victims with lesions	62	100	45	72.6	17	27.4
Male	37	100	30	81.1	7	18.9
Female	21	100	11	52.4	10	47.6
Undetermined sex	4	100	4	100	0	0

**Table 2:** Analysis of the quantitative variables identified for the entire sample

	*n*	%	Range	Mean	SD	Median	IQR
Lesions identified	560	100	1–27	9	7.1	7	3 ∼ 13
Area (cm^2^)	560	100	0.1–147.3	7.1	12.3	3	1 ∼ 7.6
Major axis (cm)	560	100	0.4–14.1	2.9	2.1	2.3	1.3 ∼ 3.6
Ratio major axis/minor axis (cm) (proxy for shape)	560	100	0.2–1	0.7	0.2	0.7	0.6 ∼ 0.8

### The lesions

**Fig. 2:**
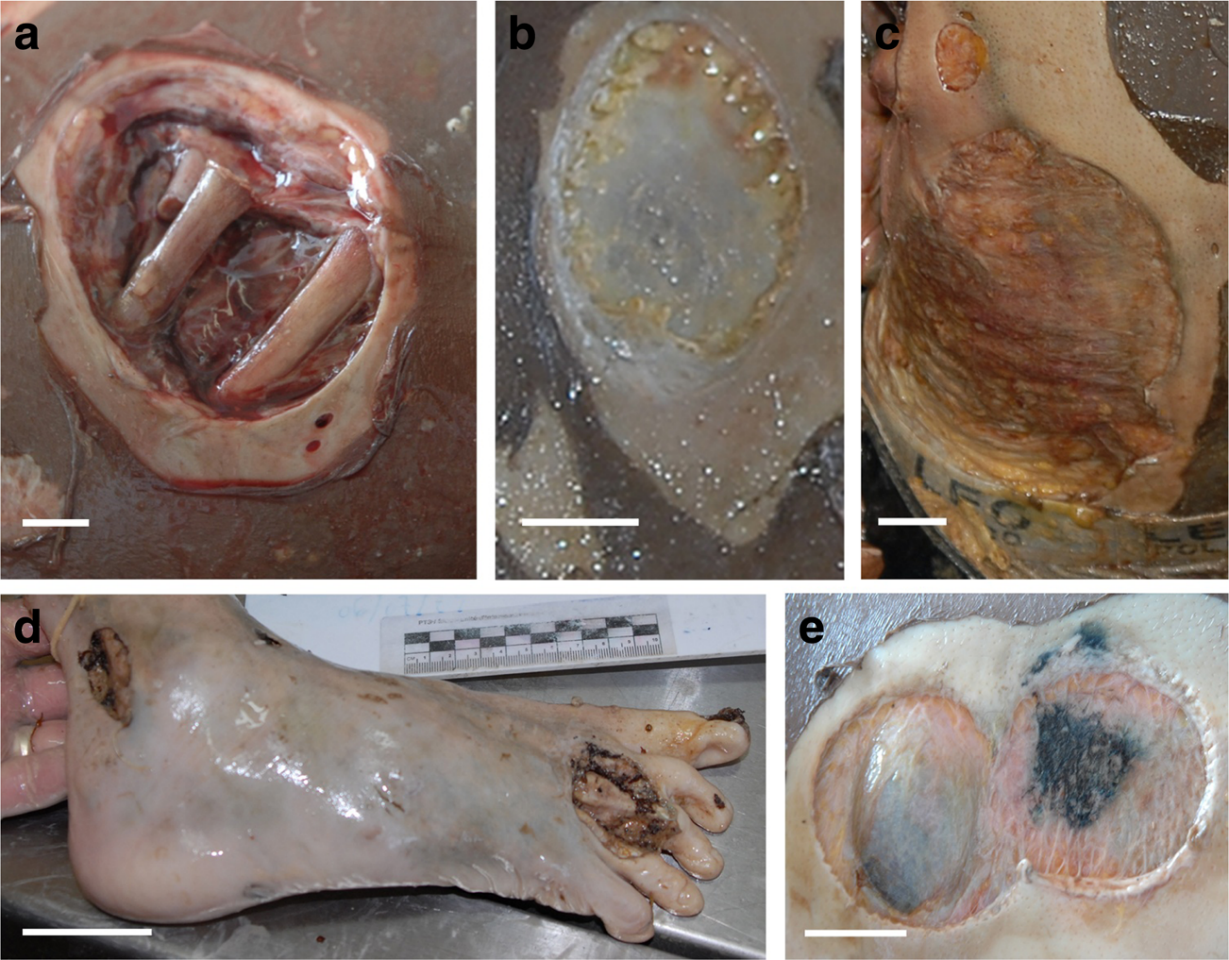
Macroscopic views of lesions found on victims of the Yemenia plane crash. Image credit: IRCGN, 2009. **a**, **b** Clearly discernable lesion. **c**–**e** Lesions of various sizes and outlines, including **e** double circular lesions. **b** and **e** Lesions showing parallel grooves all around the edges. *Scale bars* in **a** and **b**, 1 cm; in **c**, 2.5 cm; in **d**, 5 cm; in **e**, 2 cm

**Fig. 3:**
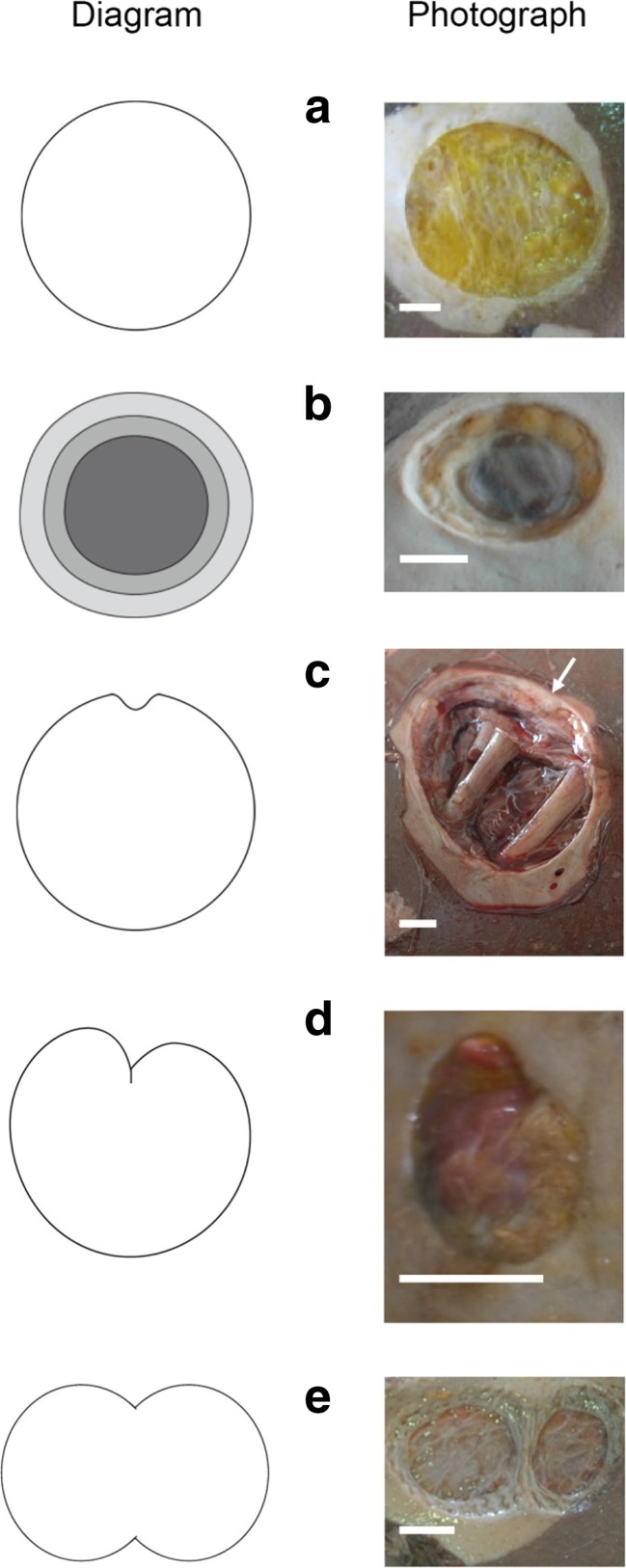
Typology of schematic variations observed in the outlines of the lesions (Adobe Illustrator software, Version CS6). **a**, **b** Circular or quasi-circular circular outline with (**a**) or without (**b**) a concave substrate. **c**, **d** Incomplete lesions with a unilateral inner triangular-shaped piece of flesh left behind (see *arrow*) (**c**), some deep enough inside the lesion to give an apple-shape form to the outline (**d**). **e** Double lesion outline, almost split into two circular or bean-shaped lesions, sometimes with a slight gap between their edges. *Scale bars* in **a**–**e**, 1 cm

The majority of lesions (89.8 %) were clearly discernable (Fig. 2a–d) while 10.2 % were superimposed and/or confluent (*χ* ^2^_1_ = 355.2, *p* < 0.001) (Fig. 2e). The presence of tooth notches on the edges of a lesion was not clearly visible, hence not possible to score for 7.9 % of lesions. Tooth notches were observed on the edges of a small number of lesions (6.8 %) (Fig. 2b, e), while most lesions (85.4 %) showed smooth and neat edges (*χ* ^2^_2_ = 682.1, *p* < 0.001). The substrate of 4.1 % of lesions could not be determined. 52.9 % of the lesions analysed presented a substrate made of fat and/or muscle (‘moderately deep’ lesions) (Fig. 2c, e) while 34.8 % of lesions occurred on tendons and ligaments, muscle and/or cortical bone (‘deep’ lesions) and 8.2 % occurred on dermis and hypodermis (‘superficial lesions’). The means of substrates were all significantly different from each other (ANOVA: *F*_2.534_ = 32.29, *p* < 0.001) (Tables 3 and 4). A great diversity of lesion outline shapes was observed (Fig. 3). Some lesions showed an almost perfectly circular outline (Fig. 3a), including some with a concave substrate (Fig. 3b), while some appeared incomplete, with a unilateral inner triangular-shaped piece of flesh left behind (Fig. 3c). In some cases, the triangular-shaped piece was deep enough inside the lesion to give an apple-shape form to the outline (Fig. 3d). Other lesions were double and looked to be almost split into two circular or bean-shaped lesions, sometimes with a slight gap between their extremities (Fig. 3e).

**Table 3:** Results of one-way ANOVA to compare area (log-transformed) and substrate of lesions

Source	SS	df	MS	F	*p*
Model	106.1	2	53	32.3	<0.001
Residual (error)	876.9	534	1.6	–	–

**Table 4:** Result of Tukey HSD (honest significant difference) to compare substrates of lesions

Pairs of substrates	Difference	Lower CI	Upper CI	*p*
Moderately deep-Superficial	1	0.5	1.5	<0.001
Deep-Superficial	1.6	1.1	2.1	<0.001
Deep-Moderately deep	0.6	0.3	0.9	<0.001

**Fig. 4:**
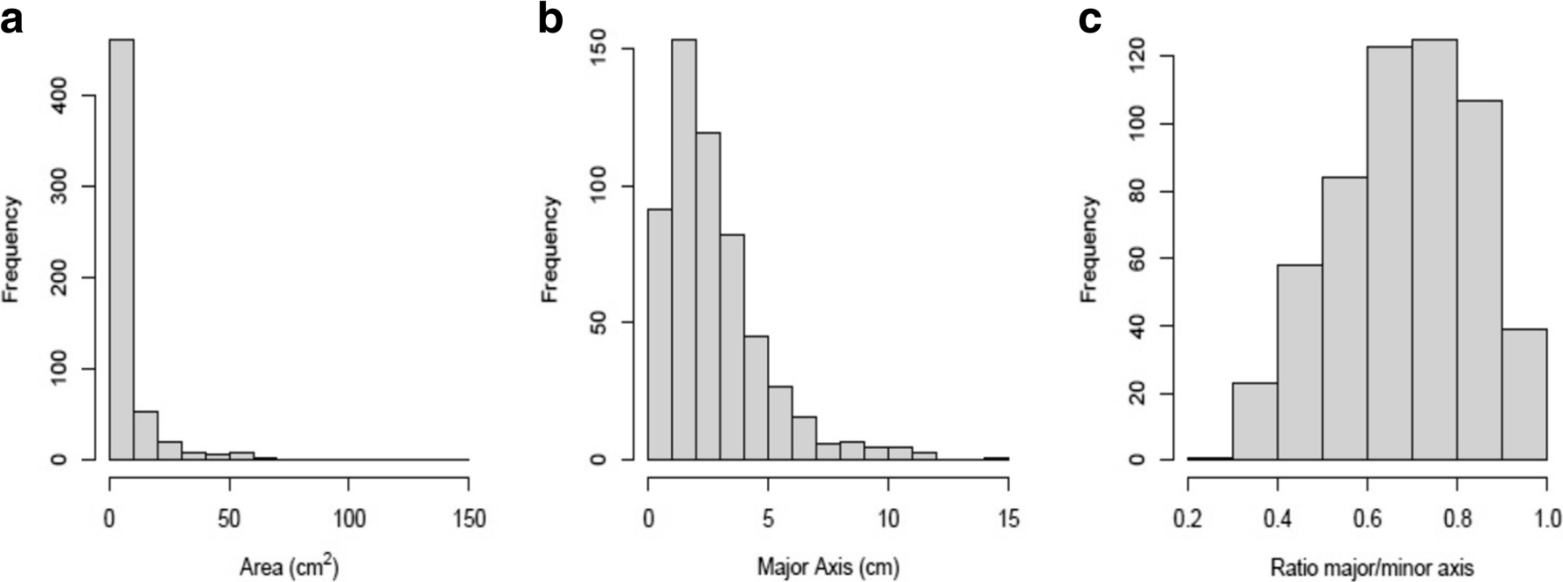
Histograms showing the distributions of area of lesions, major axis, and ratio of major axis/minor axis. The figure shows that the distributions of area of lesions, major axis, and ratio of major axis/minor axis—indicative of the shape of a lesion—are all multimodal (i.e. not normally distributed). The figure also shows descriptive statistics on macroscopic appearance of lesions. The distribution of area of lesions (**a**) ranges from 0.1 to 147.3 cm^2^ with a median of 3 cm^2^; the distribution of major axis (**b**) ranges from 0.4 to 14.1 cm with a median of 2.3 cm; the distribution of the ratio of major axis/minor axis (**c**) ranges from 0.3 to 1, with a median of 0.7

The hypothesis of a unimodal distribution of the area of the lesions was rejected (Silverman test: *p* < 0.001). The area ranged from 0.1 to 147.3 cm^2^ (IQR 1 ∼ 7.6) (Fig. 4a) with a median of 3 cm^2^ (Table 2). 82.1 % of lesions were smaller than 10 cm^2^ and 17.9 % were larger than 10 cm^2^. A correlation was identified between the area and the substrate of lesions, with the largest lesions being the deepest, on average, and the smallest lesions being the shallowest (Kendall’s Tau-b: *τ* = 0.25, *p* < 0.001); *F*_2.534_ = 32.29, *p* < 0.00; Tukey HSD: *p* < 0.001 for each pair of substrate types) (Tables 3 and 4). The distributions of major axes and perimeters of the lesions were very similar to one another, so only major axis was further analysed as it is a standard measurement in the literature on lesions [33–40]. Major axes of lesions ranged from 0.4 to 14.1 cm (IQR 1.3 ∼ 3.6) with a median of 2.3 cm (Table 2 and Fig. 4b). 98.4 % of lesions had a major axis smaller than 10 cm. The median of the distribution of the ratio of major axis/minor axis, indicative of the shape of a lesion, was 0.7 (IQR 0.6 ∼ 0.8) (Table 2 and Fig. 4c). As the hypothesis of a unimodal distribution was rejected (Silverman test: *p =* 0.04), the distribution was visually broken down into three groups of shapes to enable further analyses and comparisons with the literature: ratio of 0.2 ∼ <0.5 (‘cigar’), ratio of >0.5 ∼ <0.8 (‘ellipse’) and ratio of >0.8 ∼ 1 (‘quasi-circle or circle’), consisting in, respectively, 13, 58, and 28.9 % of the lesions. No statistically significant correlation was found between the shape and the area of lesions (Kendall’s Tau-b: *τ* = −0.02, *p* = 0.5).

## Discussion

### Identification of origin of the lesions

**Fig. 5:**
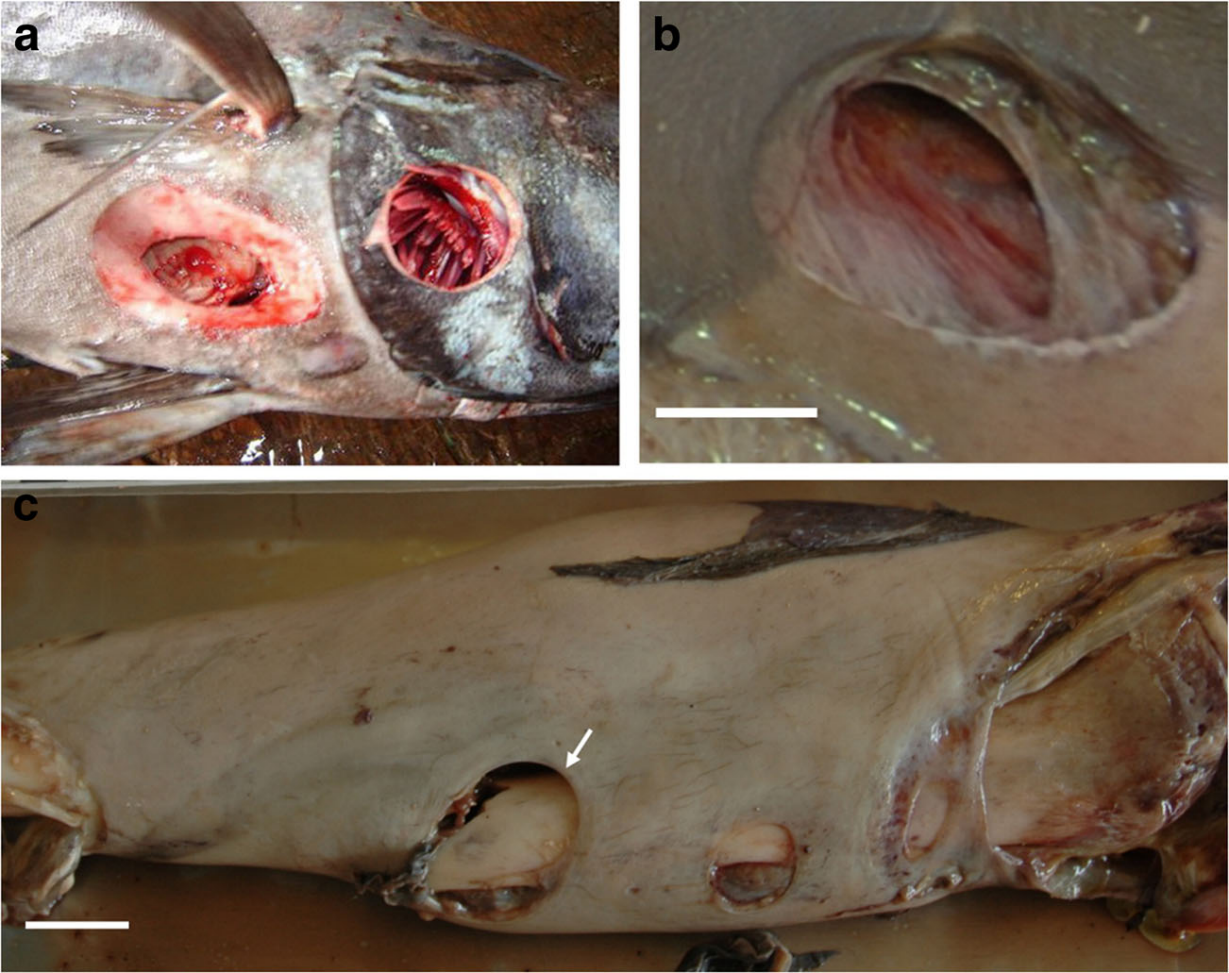
Macroscopic views of lesions. **a**, **b** Conical lesions **a** on a bony fish (image credit: Marta Eusebio, 2006) and **b** on a victim of the Yemenia plane crash (image credit: IRCGN, 2009). **c** Cookiecutter bite upon open fracture on the inferior limb (see *arrow*) of a victim of the Yemenia plane crash. Image credit: IRCGN, 2009. *Scale bars* in **b**, 1 cm; in **c**, 2 cm

Preliminary postmortem diagnosis attributed the lesions found on the victims to crabs or undetermined arthropods. The scooped out circular and neat appearance of the lesions, some showing tooth notches on the edges, suggested that they were actually bite marks, probably resulting from perimortem scavenging by marine fauna (Fig. 2b, e). A thorough literature review on marine fauna known for scavenging on human corpses or occasionally preying on them revealed that the lesions observed were (i) too large and too regular to have been punched by species of arthropods such as crabs, crawfish, sea lice and bivalve molluscs [6, 14, 17–19, 41–43] and (ii) too small, too superficial, and too circular to have been caused by large sharks whose feeding patterns are well documented [44–52]. Lesions similar to those found on the Yemenia victims are reported in the literature on a variety of marine species, alive or dead, including bony fish (i.e. swordfish and bluefin tuna), cartilaginous fish (i.e. white shark and deep-sea stingray), cetaceans (49 species) and pinnipeds (i.e. northern elephant seal and subantartic fur seal) (Fig. 5a) [26, 33, 36, 38, 39, 53–61]. The lesions described in these reports presented in the literature are identified as bite marks from ‘cookiecutter sharks’ which are said to be clearly distinctive from other animal bite marks [39, 58, 60]. Rare cases of cookiecutter bite marks on humans, alive or dead, were also reported [35, 36, 38, 62].

**Fig. 6:**
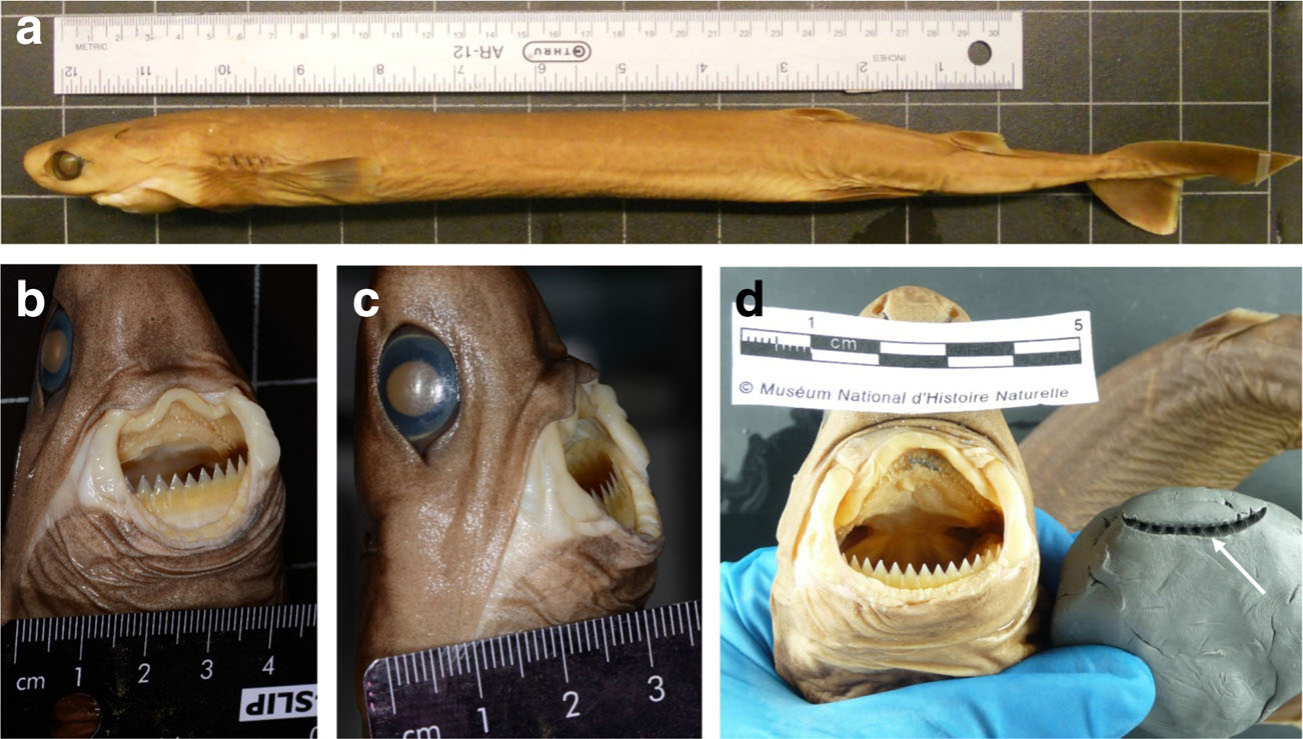
Morphology of *Isistius brasiliensis*. **a** Lateral view of a specimen from London Natural History Museum. **b**, **c** Jaws close-up showing sucker-like lips, hook-like upper teeth, and razor-sharp lower teeth, London Natural History Museum. Image credit: Patrick Campbell, 2015. **d** Reconstructed bite mark in plasticine from an *Isistius brasiliensis* specimen, Paris Natural History Museum. The *arrow* indicates the incision produced by the lower teeth of the specimen

The name ‘cookiecutter’ refers to two species of small sharks: *Isistius brasiliensis* (Quoy and Gaimard, 1824) and *Isistius plutodu*s (Garrick and Springer, 1964). While *I. plutodus* has rarely been described [38, 61, 63], *I. brasiliensis* is quite common. *I. brasiliensis* is a bioluminescent diel vertical migrator (≤3500 m deep) that inhabits tropical and oceanic waters, particularly near islands, with a high presence in Comorian and Tanzanian waters [36, 62, 64–66]. This brownish, cigar-shaped shark reaches a maximum length of 56 cm with a weight of c. 1 kg [37, 53, 65, 67] (Fig. 6a). It has a particularly distinctive mouth arranged in a transverse line with hook-like upper teeth and razor-like lower teeth surrounded by fleshy lips (Fig. 6b, c) [65]. Sucker-like lips and a modified pharynx allow cookiecutter sharks to attach to prey before the lower teeth penetrate the skin (Fig. 6d) and excise a ‘cookie’ of flesh within seconds (hence the nickname ‘cookiecutter’), then the upper teeth finally secure the piece of flesh while the shark pulls free, leaving behind a crater-like concave wound (Figs. 5a, b and 6d) [68, 69]. This peculiar feeding technique is known as ‘kleptoparasitism’ [70].

Eighty-seven percent of the lesions identified here (*n* = 487) was encompassed within the known range for the major axis of cookiecutter bite marks: 1 to 10 cm [34, 36, 53]. Whilst depth measurements were not possible from the photos of lesions used in this study, the majority (52.9 %) of lesion substrates are consistent with a moderate depth, a feature reported in the literature on cookiecutter bites on both animals and humans (1.5 to 4 cm deep) [33, 34, 38, 40]. *Isistius* spp. specimens were identified (on the underwater videos by expert ichthyologists) in the vicinity of the wreckage near Moroni. This location is compatible with the geographic distribution of the species.

In addition to these positive indications in favour of cookiecutter sharks, a comprehensive study of all other potential causes of neat circular lesions of this type confirmed the absence of other relevant plausible exterior factors, such as perimortem trauma linked with the crash, postmortem lesions of decomposition, or lesions caused at postmortem examination (i.e. caused by autopsy or DNA sampling), thereby providing further confirmation of the likelihood of cookiecutter shark bites being the most likely cause of the observed lesions.

### Observation of unreported features of cookiecutter bite marks

#### Frequency of cookiecutter bite marks

The reported range of cookiecutter bite marks on humans is of 1–7 per individual [34, 35, 40]. Here, a range of 1–27 (IQR 3 ∼ 13) bite marks was found. This difference could be explained by the difference in the size of the samples studied; here 62 victims with cookiecutter bites were analysed, while the few reports available on human victims bitten by cookiecutter sharks only deal with isolated cases [34, 35, 40]. Studies on large samples of marine animals show high frequencies of cookiecutter bites [53, 55] (i.e. 67 were recorded on a melon-headed whale [71] and 138 on a sei whale [59]).

#### Diversity in appearance of cookiecutter bite marks

The bites identified in this study showed diversity in shape, area, and depth and were often observed coexisting on one single body (Figs. 1 and 2). A similar diversity of cookiecutter bite patterns is commonly reported on marine animals (Fig. 5a) [39, 53, 72] but this is the first report on a large sample of human corpses. Double cookiecutter bites patterns (Figs. 2e and 3g) are observed here for the first time in the published literature. To the best of the authors’ knowledge, this study is the first to provide data on areas of cookiecutter bite marks. The diversity in the morphology of the bites is likely due to a combination of endogenous factors (i.e. linked to the corpses) and exogenous factors (i.e. linked to the scavengers and bias in the study material). The nature of the tissues on which the bites occurred may have accounted for some of the variations observed, depending on soft tissue characteristics (i.e. thickness, stretching, existence of previous trauma and states of decomposition) [53, 59]. The natural diversity in sizes and features of cookiecutter sharks could also contribute to the varying bite patterns, *I. brasiliensis* and *I. plutodus*, respectively, causing circular and elliptical bites [63, 73], and it is thought that mature females (the largest individuals) probably make the largest wounds [37, 53, 65, 67] while the young make the smallest superficial ones. Bites with a major axis longer than 10 cm may have been punched out by larger sharks from the Dalatiidae family, such as *Dalatias licha* (or kitefin shark), a ≤1.8 m species present in the area of the Yemenia crash and known to use kleptoparasitism as well [70]. Additionally, the position of the shark when biting could also impact the appearance of the bites [34]. Finally, in some cases the shape of the bites could have been distorted due to the camera angles used to take the autopsy photos analysed in this study.

While systematic data collection (i.e. frequency and depth of lesions) was not possible from the material used here (an issue inherent to photo-based analysis [26–28, 39, 74]), this study is the first to analyse such a large sample of human victims with such extensive cookiecutter bites (*n* = 560) and may therefore provide a more reliable picture of the diversity of cookiecutter bite marks on human corpses.

### Medicolegal problems raised by extensive cookiecutter bites

This study revealed that the presence of extensive cookiecutter bites on victims of a plane crash altered injury patterns and interfered with the ability to reconstruct events from the time of submersion. The presence of bites on tissues in advanced decomposition made distinguishing between perimortem and postmortem trauma more difficult than on fresher tissues, thus making the diagnosis of cause and manner of death and PMSI more complex. Furthermore, some bites were superimposed over pre-existing perimortem trauma, thereby concealing crucial elements from medicolegal experts (Fig. 5c). The problem of peri- and postmortem modifications caused by large sharks has been reported in the literature [44–52] but has not been addressed for small sharks such as cookiecutter sharks [59]. Due to the modifications by marine fauna interfering with the determination of the cause of death [15, 44, 45], gaining knowledge about the scavenging patterns of cookiecutter sharks on human corpses is crucial, and the scarcity of records of cookiecutter bites on humans in the literature highlights the need to identify criteria to characterise such bites.

### A novel set of identification criteria for cookiecutter bites

The key features found in this study are presented in Table 5 as a novel tool to aid in the identification of cookiecutter bites on humans. As a complementary identification tool, an initial typology derived from the data analysed in this study is proposed to better represent existing variations in the outlines of cookiecutter bite marks (Fig. 3). This set of identification criteria aims to enable similar lesions to be diagnosed more accurately in future medicolegal examinations.

**Table 5:** Positive criteria to identify cookiecutter bite marks on human corpses recovered from the ocean

Criteria	Expected ranges of values
Environmental criteria	
Geographic distribution	Between 20th parallels N and S
Depth	Surface—3 800 m
Animal criteria	
Feeding habit	Opportunistic feeder Kleptoparasitism (removal of plug of flesh)
Victim criteria	
Median number of bites	7 (IQR 3 ∼ 13)
Sexual dimorphism	Males and females equally bitten
Lesion criteria	
Shape	Elliptical or circular
Edges	Neat and sharp, possibly with tooth notches
Major axis	<10 cm
Area	<10 cm^2^
Substrate	Fat tissue or superficial layer of muscle
Depth	Moderate

**Fig. 7:**
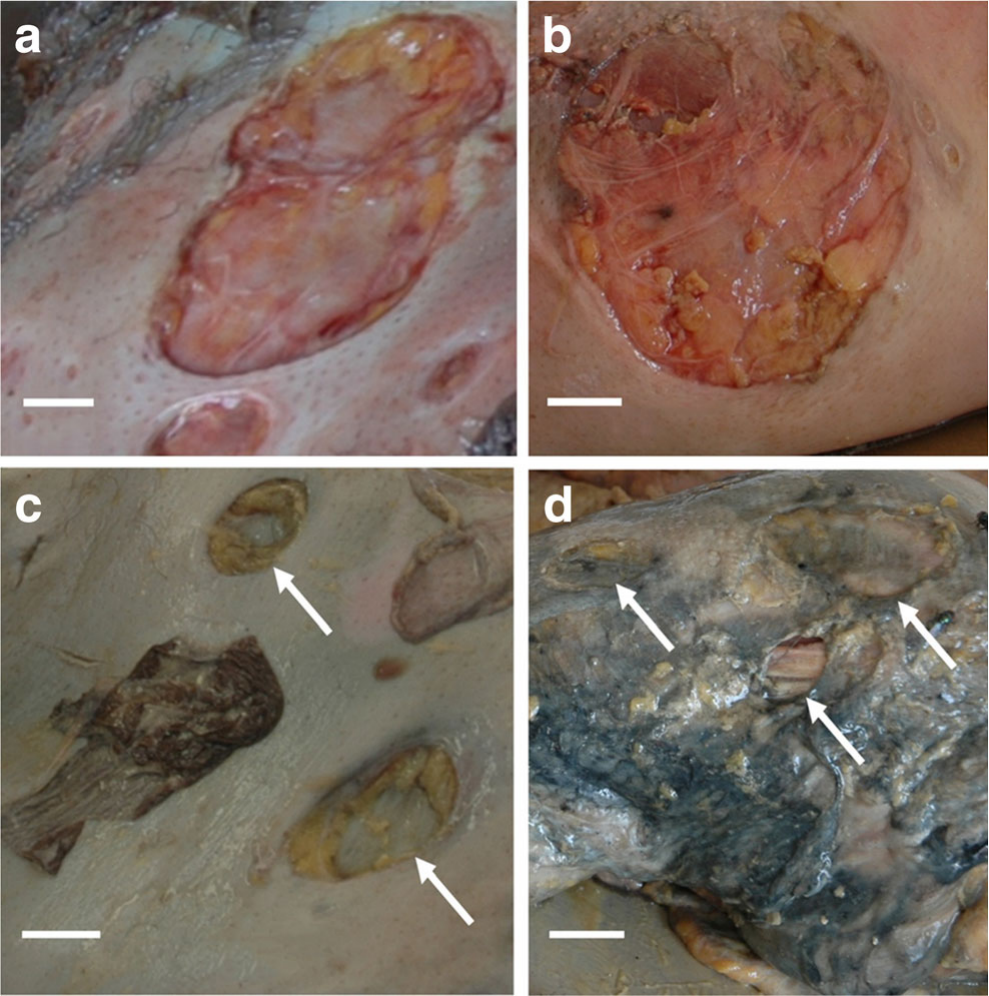
Evolution of bite marks and estimation of their age in victims of the Yemenia plane crash. Image credit: IRCGN, 2009. **a**, **b** Fresh-looking bite marks: note preserved skin on the outline and neat edges with fresh substrate. **c**, **d** Advanced-looking bite marks: note eroded outline and blunt edges with a macerated substrate (see *arrows*). *Scale bars* in **a**–**d**, 2 cm

Further diagnosis of cookiecutter bites could be provided by an estimation of the age of a bite, indicative of the period the bite occurred on the corpses. In marine biology, the age of cookiecutter bite marks on living animals is evaluated by examining the macroscopic appearance of the bites and the stage of natural healing of skin [39, 53, 59, 72]. Although this approach could not be fully applied to the corpses analysed here, identifying the appearance of the substrate (i.e. fresh or aged) and the edges (i.e. neat or blunt) permitted a partial estimation of the age of the bite marks (Fig. 7). Bite marks on the body of each victim all appeared to be the same age. Because of their different PMSIs and environments of decomposition, the corpses from the two recovery sites showed different stages of preservation. The appearance (fresh or aged) of the bites was consistent with the appearance of the surrounding soft tissues (Fig. 7a, b). Fresh bites were systematically found on bodies in early stages of decomposition, corresponding to victims recovered from Tanzania (TNZ) (PMSI up to 7 days) (Fig. 7a, b). On the other hand, aged bites were found on bodies recovered from the Comoros (CMR) (PMSI up to 60 days) that showed advanced decomposition stages (Fig. 7c, d). No recent bites were identified on degraded soft tissues. Thus, cookiecutter sharks appeared to have actively fed on the corpses only during the first week after submersion. Complementary studies will be valuable in furthering this preliminary analysis.

## Conclusion

Using a retrospective statistical and a multidisciplinary approach, this study demonstrates that the atypical circular lesions found on more than half of the victims of the Yemenia plane crash were postmortem bite marks likely caused by cookiecutter sharks (*Isistius* spp.). A total of 560 cookiecutter bite marks were identified, a sample far larger than other samples available in the published literature. The methodology applied here to a large sample expands the understanding of cookiecutter bite patterns on human corpses and shows that the importance of cookiecutter sharks as taphonomic agents in marine environments may have been previously underestimated. The high frequency of cookiecutter bite marks, including some occasionally superimposed over pre-existing trauma, led to difficulties in identifying perimortem trauma, an understanding of which is essential in legal medicine to determine the cause and manner of death. To address this issue, this study provides a new set of measurements and macroscopic traits to identify cookiecutter bite marks in future forensic cases of victims recovered from marine environments to assist in the reconstruction of events around the time of death. As a complement, standardised protocols for comprehensive data collection at postmortem examination, including autopsy photographs and descriptions of atypical lesions, could helpfully be designed to reduce the risk of uncertainty in the identification between perimortem and postmortem trauma and also allow non-invasive forensic retrospective research.

## Abbreviations

ANOVA: Analysis of variance
CI: Confidence interval
CMR: Comoros
DNA: Deoxyribonucleic acid
HSD: Honest significant difference
ICC: Intra-class correlation coefficient
IQR: Interquartile range
MS: Mean square
PMSI: Postmortem submersion interval
SD: Standard deviation
SS: Sum of squares
TNZ: Tanzania
*χ*^2^: Chi-square
